# Knowledge and Attitudes Towards ECG Interpretation Among Intensive Care Nurses: A Greek Cross‐Sectional Study

**DOI:** 10.1111/nicc.70505

**Published:** 2026-04-25

**Authors:** Lampros Skaliaris, George Kipourgos, Evangelia Andreopoulou, Angeliki Gkotsi, Eleni Albani, Anastasios Tzenalis

**Affiliations:** ^1^ Intensive Care Unit European Interbalkan Medical Center Thessaloniki Greece; ^2^ Surgical Cardiac Intensive Care Unit, Department of Cardiac Surgery University of Patras Patras Greece; ^3^ Haemodynamic Laboratory, Department of Cardiology University of Patras Patras Greece; ^4^ Nursing Department University of Patras Patras Greece

**Keywords:** clinical competence, critical care education, ECG interpretation, intensive care nursing, patient safety

## Abstract

**Background:**

Timely recognition of cardiac arrhythmias is essential in Intensive Care Units (ICUs). Electrocardiogram (ECG) interpretation is a core component of nursing practice, yet international studies show persistent gaps. In Greece, limited evidence exists regarding ICU nurses' interpretation, educational preparedness and role perception in ECG interpretation.

**Aim:**

The aim of this study was to evaluate ICU nurses' knowledge, attitudes and self‐perceived preparedness regarding ECG interpretation and to investigate whether these outcomes were associated with demographic and professional characteristics.

**Study Design:**

A descriptive, cross‐sectional web‐based survey was conducted among ICU nurses in Greece between October 2024 and February 2025. The instrument included demographic variables, 10 true/false knowledge items and 10 Likert‐scale attitude items. Internal consistency was acceptable (α = 0.81–0.87). Data analysis included descriptive statistics, chi‐squared tests and multivariable logistic regression (*p* < 0.05).

**Results:**

A total of 100 ICU nurses participated in the study. The mean knowledge score was 6.1 ± 1.8/10, with high accuracy on basic items (88%) but low performance on complex patterns (24%). Prior ECG training was the only independent predictor of adequate knowledge (OR = 2.85, 95% CI: 1.12–7.26, *p* = 0.028). The mean attitude score was 38.4 ± 6.2/50; although 96% recognised ECG interpretation as essential, 49% reported limited knowledge. Positive attitudes were linked to prior training, ICU experience and frequency of ECG evaluation, whereas 25% expressed ambiguity about whether ECG interpretation falls within nursing responsibilities.

**Conclusions:**

ICU nurses demonstrated moderate theoretical ECG knowledge but variable preparedness and role clarity. Prior training, ICU type and clinical experience were key determinants. Standardised, simulation‐based training and clearer responsibility delineation could strengthen cardiac monitoring safety.

**Relevance to Clinical Practice:**

Targeted ECG education and explicit clarification of nurses' responsibilities in cardiac monitoring are essential to strengthen arrhythmia recognition and timely intervention in ICUs. Implementing structured training pathways and ongoing assessment of ECG interpretation skills may support safer cardiac monitoring practices and more autonomous, confident intensive care nursing.

## Impact Statements

- What is known about the topic?
  ○ ECG interpretation is essential for ICU nurses and is critical for timely arrhythmia recognition and prevention of clinical deterioration.
  ○ International studies have reported persistent gaps in nurses' ECG interpretation skills, particularly for complex rhythms.
  ○ Access to structured ECG training and supportive organisational culture varies widely across countries and institutions.
- What this paper adds?
  ○ Provides the first national data on ECG interpretation knowledge and preparedness among ICU nurses in Greece.
  ○ Demonstrates that prior ECG training, ICU type and years of experience are key determinants of interpretation, whereas gender and educational background are not.
  ○ Highlights the need for mandatory in‐service ECG training, simulation‐based practice and clearer role delineation to improve diagnostic readiness and patient safety in ICUs.

## 1 Introduction

Timely recognition of cardiac arrhythmias is critical for improving survival and reducing mortality among patients admitted to Intensive Care Units (ICUs). Electrocardiography (ECG) remains an essential tool for monitoring cardiac rhythm and detecting electrical or structural abnormalities of the myocardium [1]. Continuous ECG monitoring is widely recognised as a core nursing practice for identifying arrhythmias, acute ischaemic events and disturbances in electrolyte or acid–base balance [2]. In high‐acuity environments such as ICUs, rapid interpretation of rhythm changes is fundamental, as delays are directly linked to preventable clinical deterioration and adverse outcomes.

Despite the incorporation of smart monitoring systems and automated alert algorithms, ECG interpretation in ICUs continues to require advanced clinical judgement and experience, especially in complex and rapidly evolving care situations [3]. Technological innovations do not replace nursing expertise; instead, they amplify the need for sophisticated ECG interpretation and continuous education [4]. ICU nurses function as front‐line decision‐makers in the early detection of cardiac complications and in the prompt initiation of life‐saving interventions [5]. Within international clinical frameworks and clinical guidelines, the ability to accurately interpret abnormal ECG findings is considered a fundamental and non‐negotiable intensive care nursing skill.

### 1.1 Background

Although ECG interpretation is integral to safe ICU care, a growing body of evidence highlights persistent gaps in nurses' ECG knowledge and interpretive accuracy. A systematic review by Chen et al. [6] demonstrated frequent inaccuracies in rhythm recognition, particularly in high‐acuity areas, whereas Ng and Christensen [7] found that even experienced ICU nurses may misinterpret complex arrhythmias. Such deficits have been associated with delayed interventions and reduced patient safety [5]. Structured education programmes, blended learning and simulation‐based approaches have been shown to enhance ECG interpretation [8, 9], yet implementation varies significantly across institutions and countries.

Despite this international interest, little is known about ECG interpretation among ICU nurses in Greece. This knowledge gap is particularly relevant given the heterogeneous educational backgrounds of the national nursing workforce spanning university graduates and Technological Educational Institutes, each with differing emphases on theoretical knowledge and applied ECG interpretation centred organisation of the national healthcare system may contribute to role ambiguity, meaning uncertainty regarding the scope of nursing responsibilities in clinical tasks such as ECG interpretation [10]. Understanding the influence of these factors within the ICU context is essential for informing national and European efforts to develop standardised frameworks for ECG interpretation training and practice in intensive care nursing.

### 1.2 Purpose of the Research

The aim of this study was to evaluate ICU nurses' knowledge, attitudes and self‐perceived preparedness regarding ECG interpretation and to investigate whether these outcomes were associated with demographic and professional characteristics.

## 2 Design and Method

### 2.1 Design of Research

This study was conducted and reported in accordance with the Strengthening the Reporting of Observational Studies in Epidemiology (STROBE) guidelines. A descriptive, cross‐sectional, web‐based survey design was employed. Data were collected between October 2024 and February 2025. The primary aim was to assess ICU nurses' knowledge, attitudes and self‐perceived preparedness regarding ECG interpretation.

### 2.2 Sample and Participation Criteria

Τhe study sample consisted of registered nurses employed in adult ICUs in Greece. Inclusion criteria required participants to be currently employed in an adult ICU and to hold at least a tertiary‐level nursing qualification (Technological Educational Institute or University degree). Exclusion criteria comprised secondary‐level nursing graduates, as well as nursing students and interns.

A non‐probability convenience sampling strategy was used. The online questionnaire was distributed through professional nursing networks, social media platforms and email lists. As the survey link was openly accessible, the total number of nurses approached and the exact response rate could not be determined, which represents a methodological limitation.

Given the exploratory nature of the study, no formal a priori sample size calculation was conducted. However, a post hoc power analysis was conducted using the G*Power software (version 3.1.9.7). Given that the primary inferential analyses involved chi‐squared tests of independence, the power calculation was based on these tests. Given the achieved sample size, an alpha error probability of 0.05, and degrees of freedom ranging from 1 to 4, the analysis confirmed that the study achieved adequate statistical power (1—β > 0.80) to detect medium‐to‐large effect sizes (equivalent to an effect size w or Cramer's V ≥ ). This limitation regarding the lack of an a priori sample size calculation is acknowledged, and subgroup analyses should be interpreted with caution.

### 2.3 Data Collection Tool

The survey instrument was adapted from Buluba et al. [11] and comprised three sections with a total of 26 items: (a) 6 items on demographics and professional characteristics, (b) 10 true/false items assessing theoretical knowledge of cardiac electrophysiology and arrhythmias and (c) 10 items assessing attitudes on a 5‐point Likert scale. For the knowledge section, a score of 1 was given for each correct answer and 0 for incorrect answers, yielding a maximum total score of 10. A cut‐off score of ≥ 7/10 (70%) was used to indicate ‘adequate knowledge’, consistent with previous literature using this tool. It is important to note that the knowledge items assessed conceptual recall rather than practical interpretation of ECG strips. Scenarios, vignettes or actual ECG rhythm strips were not used in this study, as the primary aim was to evaluate foundational theoretical knowledge and self‐perceived preparedness, which serve as a baseline before assessing clinical interpretative skills.

The instrument underwent forward–backward translation and cultural adaptation into Greek language. Content validity was established through expert review and cognitive debriefing with 15 ICU nurses. Internal consistency was satisfactory (Cronbach's α = 0.81 for knowledge items; 0.87 for attitude items). Although face and content validity were supported, construct validity (EFA/CFA) and test–retest reliability were not assessed, and this is recognised as a limitation. Composite scores for the knowledge and attitude subscales were calculated to support comprehensive interpretation.

Data were collected via an online survey hosted on the Google Forms platform. The questionnaire was designed to be concise, taking approximately 5 to 10 min to complete. To prevent missing data, all items in the online form were marked as ‘mandatory’, ensuring that participants could not submit incomplete responses. Additionally, the platform's ‘limit to one response’ feature was enabled to prevent duplicate submissions. Regarding the attitude items (Likert scale), the anonymity of the web‐based design helped mitigate social desirability bias, although central tendency bias remains an inherent limitation of Likert scales.

### 2.4 Data Analysis

Data were analysed using SPSS v26. Normality of continuous variables (e.g., composite knowledge and attitude scores) was assessed using the Shapiro–Wilk test and visual inspection of histograms. Since the composite scores deviated from a normal distribution, descriptive statistics for these nonparametric data would ideally be represented by medians and interquartile ranges (IQR). However, for ease of interpretation and comparison with similar international studies, we primarily present mean and standard deviation (SD). It is crucial to note that the inferential tests (*p* values) presented in our tables were not derived from comparing these continuous means; rather, associations between categorical variables (e.g., adequate vs. inadequate knowledge based on the ≥ 7/10 cut‐off) and professional characteristics were tested using the chi‐squared test of independence. Effect sizes for these categorical comparisons are reported using the Phi coefficient or Cramer's V. Multivariable logistic regression analyses were conducted to adjust for potential confounders. The significance level was set at *p* < 0.05.

### 2.5 Ethical Considerations

The study complied with the Declaration of Helsinki and the EU General Data Protection Regulation (GDPR, Regulation 2016/679). Ethics approval was obtained from the Research Ethics and Deontology Committee of the Department of Nursing, University of Patras, Greece (Approval No.: PN.10/09/24–3311). Participation was voluntary, and electronic informed consent was obtained prior to survey completion. No personal identifiers were collected. Data were stored in encrypted files on secure servers with restricted access and will be retained for 5 years. A data availability statement is provided to ensure transparency.

## 3 Results

A total of 100 ICU nurses participated in the study. Most participants were female (62%), and nearly half were aged 20–29 years (47%). The majority worked in general ICUs (54%), followed by cardiac (25%) and surgical ICUs (19%). Regarding clinical experience, 43% reported up to 5 years of professional practice, whereas 14% had more than 20 years. Two‐thirds (64%) had previously attended ECG‐related training, and almost all respondents (97%) expressed a strong interest in further ECG education (Table 1).

**TABLE 1:** Demographic and professional characteristics of ICU nurses (*n* = 100).

Variable	*n*	%
Age		
20–29	47	47.0
30–39	27	27.0
40–55	26	26.0
Gender		
Male	38	38.0
Female	62	62.0
ICU type		
General ICU	54	54.0
Emergency ICU	1	1.0
Neurosurgical ICU	1	1.0
Surgical ICU	19	19.0
Cardiac ICU	25	25.0
Years of total clinical experience		
< 1 year	5	5.0
1–5 years	43	43.0
6–10 years	20	20.0
11–20 years	18	18.0
> 20 years	14	14.0
Years of ICU experience		
< 1 year	13	13.0
1–5 years	56	56.0
6–10 years	17	17.0
11–20 years	6	6.0
> 20 years	8	8.0
Educational level		
Technological degree (TEI)	37	37.0
University degree	32	32.0
Postgraduate degree	29	29.0
Doctoral degree	2	2.0
Work position		
Head nurse	1	1.0
Senior nurse	11	11.0
Clinical instructor	0	0.0
Staff nurse	88	88.0

The median composite knowledge score was 6.0 (IQR: 2.0) of 10 (Cronbach's α = 0.81). Performance was highest on basic electrophysiology items, such as identifying ventricular depolarisation (88% correct), whereas accuracy on more complex items was substantially lower, including questions related to wave sequence (24% correct) (Table 2). Prior ECG training was significantly associated with higher composite knowledge scores (*p* = 0.009, Cramer's V = 0.26) indicating a moderate effect size, suggesting that training may have a meaningful impact on nurses' theoretical ECG knowledge. Cardiac ICU nurses demonstrated better performance on specialist items, such as recognition of Mobitz type II atrioventricular block (*p* = 0.002, Phi = 0.32) representing a moderate association, which may reflect the greater exposure to complex arrhythmias in cardiac ICU settings. Similarly, nurses with more than 20 years of experience more frequently identified pathological Q waves as indicative of previous myocardial infarction (92.9%; *p* = 0.024, Phi = 0.27). No consistent associations were observed with educational level, although Technological Educational Institute graduates performed better on selected practical items compared with university‐trained colleagues (Table 3).

**TABLE 2:** ICU nurses' knowledge of ECG interpretation (*n* = 100).

Item (True/False)	Correct *n* (%)	Incorrect *n* (%)
QRS complex represents ventricular depolarisation	88 (88.0)	12 (12.0)
If P wave is absent, atrial conduction issue	80 (80.0)	20 (20.0)
Pathological Q waves indicate previous myocardial infarction	73 (73.0)	27 (27.0)
PR interval not constant in Mobitz II AV block	74 (74.0)	26 (26.0)
ECG can detect left ventricular hypertrophy (LVH)	74 (74.0)	26 (26.0)
Atrial flutter = regular rhythm with stable R‐R	64 (64.0)	36 (36.0)
Ventricular fibrillation shows unrecognisable P/T waves but recognisable QRS	49 (49.0)	51 (51.0)
P wave represents repolarisation of atria	33 (33.0)	67 (67.0)
Correct sequence of ECG waves (T, P, QRS, PR, ST, U)	24 (24.0)	76 (76.0)
Atrial fibrillation could be considered a normal rhythm	19 (19.0)	81 (81.0)

*Note:* Composite knowledge score (10 items): Median = 6.0, IQR = 2.0 (Cronbach's α = 0.81). Items are ordered by percentage of correct responses (from highest to lowest).

**TABLE 3:** Associations between ECG knowledge scores and professional characteristics (*n* = 100).

Characteristic	Mean knowledge score (±SD)	Test statistic	*p*	Effect size
ICU Type (General vs. Cardiac)	5.7 ± 1.9 vs. 6.6 ± 1.6	χ^2^ = 9.952	0.002	Phi = 0.32
Prior ECG training (Yes vs. No)	6.8 ± 1.7 vs. 5.4 ± 1.8	χ^2^ = 6.836	0.009	V = 0.26
Years of experience (> 20 years)	6.9 ± 1.5 vs. 5.9 ± 1.9	χ^2^ = 11.240	0.024	Phi = 0.27
Educational level (TEI vs. Univ)	6.4 ± 1.7 vs. 5.8 ± 1.9	χ^2^ = 4.807	0.088	n.s.
ECG use frequency	6.1 ± 1.8 (all categories)	χ^2^ = 5.874	0.118	n.s.

*Note:* The Mean Knowledge Score (±SD) is presented purely for descriptive purposes. The Test Statistic (χ^2^), *p*‐value, and Effect Size correspond to the Chi‐square test of independence performed on the dichotomised categorical variable (Adequate Knowledge ≥ 7/10 vs. Inadequate Knowledge < 7/10).

To further explore predictors of ECG knowledge, a multivariable logistic regression model was constructed using ‘adequate knowledge’ (≥ 7/10 correct answers) as the dependent variable. After adjusting for age, gender, education, ICU type, years of experience and prior ECG training, only prior ECG training remained a significant independent predictor (OR = 2.85, 95% CI: 1.12–7.26, *p* = 0.028). A secondary model examining attitudes towards ECG interpretation showed that prior ECG training (OR = 3.41, 95% CI: 1.18–9.85, *p* = 0.023) and < 10 years of experience (OR = 2.16, 95% CI: 1.01–4.64, *p* = 0.047) were associated with reporting insufficient preparedness. No significant effects were found for gender or educational level in either model (Table 4).

**TABLE 4:** Multivariable logistic regression analysis of predictors of ECG knowledge and attitudes among ICU nurses.

Predictor variable	Adequate knowledge (≥ 7/10) OR (95% CI)	*p*	Inadequate preparedness (Attitudes) OR (95% CI)	*p*
Age (≥ 40 vs. < 40 years)	1.18 (0.55–2.54)	0.67	0.94 (0.48–1.86)	0.87
Gender (Female vs. Male)	0.92 (0.43–1.97)	0.84	1.08 (0.52–2.24)	0.82
Education (TEI vs. AEI)	1.25 (0.59–2.64)	0.56	0.98 (0.47–2.05)	0.95
ICU Type (Cardiac vs. General/Surgical)	1.42 (0.66–3.05)	0.36	1.67 (0.78–3.56)	0.19
Experience (> 20 years vs. ≤ 20)	1.38 (0.60–3.16)	0.45	0.82 (0.37–1.84)	0.64
Experience (< 10 years vs. ≥ 10)	1.05 (0.50–2.20)	0.89	2.16 (1.01–4.64)	0.047*
Prior ECG Training (Yes vs. No)	2.85 (1.12–7.26)	0.028*	3.41 (1.18–9.85)	0.023*

*p < 0.05.

The median composite attitude score was 34.0 (IQR: 4.75) of 50 (Cronbach's α = 0.87). Almost all respondents (96%) agreed that ECG interpretation is essential for arrhythmia recognition. However, 49% reported limited knowledge, and half (50%) considered their previous training insufficient (Table S1). Cardiac ICU nurses were more likely to perceive themselves as inadequately prepared compared with general ICU nurses (40% vs. 22%; *p* = 0.010, Cramer's V = 0.29). Nurses who frequently evaluated ECGs in their daily practice were more likely to consider interpretation an essential nursing responsibility (*p* < 0.001, Cramer's V = 0.35). Longer ICU experience was associated with greater perceived difficulty in ECG interpretation and higher recognition of training needs (*p* = 0.010–0.020, Cramer's V = 0.28–0.29). Nurses in general ICUs more often prioritised vital signs monitoring over ECG interpretation compared with those in cardiac ICUs (*p* = 0.022, Phi = 0.25). Less experienced nurses (< 10 years) were also more likely to report lower self‐perceived preparedness (*p* = 0.027, Phi = 0.21). Additionally, 25% expressed neutrality or disagreement with ECG interpretation as part of nursing responsibilities, indicating uncertainty regarding professional role boundaries. Prior ECG training was consistently associated with more positive attitudes: All trained nurses (100%) acknowledged its importance compared with 88.9% of untrained nurses (*p* = 0.025, Cramer's V = 0.25) (Table S2).

Overall, both knowledge and attitudes were strongly influenced by prior ECG training, ICU type and years of professional experience. No significant associations were found with gender or educational level. A summary of the significant associations is presented in Table S3. Effect sizes were reported alongside *p* values to support interpretation of practical significance. Adjustments for multiple testing were not performed, as the study was exploratory and aimed at identifying potential associations rather than confirmatory hypothesis testing.

## 4 Discussion

This study examined ICU nurses' ECG interpretation knowledge and self‐perceived preparedness within a national framework. Overall, participants demonstrated moderate theoretical knowledge, with a median score of 6.0 (IQR: 2.0) of 10 and variable attitudes regarding role responsibility and training needs. These findings align with international evidence indicating that, although ICU nurses are expected to play a central role in arrhythmia detection, ECG interpretation frequently remains below optimal levels [6, 7, 11]. However, it should be noted that the present study assessed theoretical ECG knowledge and self‐perceived preparedness rather than direct observation of ECG interpretation performance; therefore, the findings should be interpreted as reflecting nurses' cognitive understanding and perceived readiness rather than objective ECG interpretation performance. Similar observations have been reported in Middle Eastern and European settings, where nurses' ECG knowledge, perceived preparedness and ECG interpretation performance are influenced by institutional culture, perceived support and local scope‐of‐practice norms [12, 13].

A prominent finding was the discrepancy between good performance on basic electrophysiology items and significantly lower accuracy on more complex concepts. Although 88% correctly identified the QRS complex, fewer than one in four identified the correct wave sequence. This reinforces the distinction between theoretical recall and practical ECG interpretation—an issue frequently highlighted in ECG education literature [8, 9]. The consistently low performance on complex patterns underscores the need for experiential learning methods such as rhythm‐strip interpretation, simulation‐based drills and supervised case‐based practice. Prior research shows that these strategies enhance diagnostic speed, pattern recognition and decision‐making confidence—skills that are crucial in high‐acuity ICU environments where cognitive load, stress and interruptions can hinder performance [14].

Role perception emerged as an equally significant issue, with a quarter of nurses expressing neutrality or disagreement about ECG interpretation as part of nursing responsibilities. This highlights role ambiguity in Greece ICUs, likely influenced by hierarchical, physician‐centred models and unclear job descriptions. Previous national studies have documented similar ambiguity, noting overlapping responsibilities and limited nursing autonomy in cardiac monitoring [15]. Comparable findings across Europe suggest that organisational culture (referring to the shared norms, values and expectations within healthcare teams) and managerial expectations substantially shape professional identity, perceived interpretation ability and nurses' willingness to assume advanced clinical responsibilities [12, 13]. These parallels imply that institutional climate plays a pivotal role in determining whether ICU nurses feel responsible and adequately empowered to undertake complex interpretive tasks.

Prior ECG training consistently emerged as a decisive predictor of both knowledge and attitudes, reinforcing the value of structured educational initiatives. Trained nurses not only scored higher but also expressed more positive attitudes towards ECG interpretation. This echoes international evidence demonstrating that simulation‐based ECG training, blended learning and structured ECG interpretation curricula substantially improve accuracy and confidence [8, 9]. In Greece, where standardised ECG training in both undergraduate and continuing education pathways remains fragmented, implementing mandatory, structured in‐service modules could be a cost‐effective strategy to enhance patient safety and improve arrhythmia detection. Comparable models in the United Kingdom and Scandinavia—where ECG interpretation training is incorporated into continuing professional development—may serve as useful frameworks.

The importance of educational preparedness was further supported by associations between attitudes, emotional intelligence and self‐efficacy documented in international literature. Studies have shown that emotional intelligence and critical thinking reinforce clinical confidence, whereas a strong professional self‐concept predicts more effective clinical decision‐making [13, 16]. These constructs may be particularly relevant for ECG interpretation, a high‐stakes task requiring calm cognitive processing and accurate pattern recognition under pressure.

Multivariable analyses confirmed that prior ECG training was the strongest independent predictor of adequate knowledge and positive attitudes, even after adjusting for demographic and professional variables. Less experienced nurses (< 10 years) were more likely to report insufficient preparedness, suggesting that clinical exposure alone cannot compensate for gaps in structured training. These findings support the argument that policy‐level educational reforms—rather than experience‐based learning alone—are necessary to ensure consistent ECG interpretation across ICUs. Similar patterns have been seen in studies on evidence‐based practice readiness, where the quality of educational exposure strongly influences perceived ECG interpretation ability and professional confidence [13].

Interpretation of associations must be cautious. Differences by ICU type and experience may reflect unmeasured factors such as recency of graduation, undergraduate curriculum variation and daily exposure to arrhythmias. Furthermore, cardiac ICU nurses' higher reports of perceived insufficiency may reflect heightened awareness of clinical complexity rather than lower ECG interpretation ability. Attitudes were self‐reported and therefore subject to response bias, although perceptions remain meaningful indicators of professional role identity and learning needs. As suggested by recent literature, institutional culture, emotional intelligence and self‐concept play substantial roles in shaping how ECG interpretation ability is perceived [13, 17].

In summary, this study provides context‐specific evidence that ICU nurses in Greece demonstrate moderate ECG knowledge and variable preparedness, with ECG interpretation ability strongly shaped by prior training, ICU environment and professional experience. Addressing these gaps requires structured educational interventions, role clarification and organisational policies that promote continuous development of ECG interpretation skills. Integrating emotional intelligence training, reflective practice and supportive institutional culture—elements highlighted across international research—may further strengthen diagnostic confidence and professional readiness among ICU nurses in Greece [16, 17].

### 4.1 Limitations

This study has several limitations that should be considered when interpreting the findings. First, the use of a convenience, web‐based sampling strategy limits generalisability, as the true response rate could not be calculated and nurses with greater interest in ECG interpretation may have been more likely to participate. Second, ECG knowledge was assessed using true/false items reflecting theoretical understanding rather than interpretation of actual ECG rhythm strips. Binary responses such as these have a 50% guessing probability, which may overestimate the participants' actual knowledge levels compared to multiple‐choice questions or practical scenario‐based assessments. Although this approach allowed for rapid assessment across a large sample, it may not fully capture applied ECG interpretation, and performance may differ in real‐time clinical situations.

Furthermore, attitudes and preparedness were self‐reported and therefore subject to recall or social desirability bias. Cardiac ICU nurses' higher perceived insufficiency, for example, may reflect heightened awareness of clinical complexity rather than lower ECG interpretation ability. Finally, variation in undergraduate curricula and continuing education opportunities across Greece may have contributed to between‐group differences that were not fully captured by the demographic variables included.

Despite these limitations, the study provides important context‐specific insights into ECG interpretation ability among ICU nurses in Greece and highlights priority areas for targeted educational and organisational interventions.

## 5 Implications for Clinical Practice

The findings of this study highlight several important implications for clinical practice in ICUs. First, the moderate theoretical knowledge and substantial gaps in applied ECG interpretation skills reinforce the need for structured, mandatory in‐service ECG education tailored to ICU nursing roles. Simulation‐based learning, hands‐on rhythm‐strip workshops and assessment of ECG interpretation skills should be integrated into routine clinical training to enhance diagnostic accuracy and decision‐making in time‐critical situations. Second, the strong association between prior ECG training and both knowledge and preparedness underscores the value of ongoing professional development; institutions should prioritise protected time and accessible training pathways for ICU nurses. Third, the observed variability in role perception suggests a need for clearer organisational definitions of nursing responsibilities in cardiac monitoring, ensuring that nurses feel empowered to recognise arrhythmias and initiate appropriate escalation. Finally, standardising ECG interpretation expectations across ICUs may improve consistency of care, reduce delays in intervention and ultimately enhance patient safety. Incorporating structured ECG education into onboarding programmes, mentorship models and regular assessment of ECG interpretation skills could support safer clinical practice and more confident, autonomous intensive care nursing.

## 6 Conclusion

This study provides novel, context‐specific insights into ICU nurses' ECG interpretation knowledge and preparedness in Greece. Although participants demonstrated moderate theoretical knowledge, notable gaps remained in applied interpretation skills, as well as in role perception and self‐reported preparedness. Prior ECG training, ICU type and years of clinical experience emerged as key determinants of ECG interpretation ability, whereas gender and educational background showed no consistent influence.

Addressing these gaps requires a dual approach involving targeted educational interventions—such as mandatory in‐service ECG training, simulation‐based practice, and structured ECG interpretation assessments—and organisational strategies that clarify nurses' professional responsibilities in cardiac monitoring. Policies promoting continuous development of ECG interpretation skills are essential to strengthen diagnostic readiness, enhance patient safety and align national ICU practice with international standards.

Future research should include multicentre studies, incorporate validated psychometric tools and use direct rhythm‐strip interpretation tasks to more accurately evaluate applied ECG interpretation performance. Implementing these strategies could contribute to safer cardiac monitoring practices and improved clinical outcomes across ICUs in Greece.

## Funding

The authors have nothing to report.

## Ethics Statement

Ethics approval was obtained from the Research Ethics Committee of the University of Patras, Greece (Approval No. RN.10/09/24–3311, approved on 10 September 2024).

## Consent

The authors have nothing to report.

## Conflicts of Interest

The authors declare no conflicts of interest.

## Supporting information


**Table S1:** ICU Nurses' attitudes towards ECG interpretation (*n* = 100).

**Table S2:** Significant associations between ICU nurses' attitudes towards ECG interpretation and professional characteristics (*n* = 100).

**Table S3:** Summary of significant associations between ECG knowledge and attitudes and professional characteristics (*n* = 100).

## Data Availability

The data that support the findings of this study are available on request from the corresponding author. The data are not publicly available due to privacy or ethical restrictions.
