# Supplementary material for: Knowledge and Attitudes Towards ECG Interpretation Among Intensive Care Nurses: A Greek Cross‐Sectional Study
Source: Nurs Crit Care. 2026 Apr 25;31:e70505. doi: 10.1111/nicc.70505 (PMC13109745; doi:10.1111/nicc.70505)
Supplement: Supplementary file 1 — Table S1: ICU Nurses' attitudes towards ECG interpretation (n = 100). [file NICC-31-0-s002.docx]

# Supplementary Tables

# Table S1. ICU Nurses’ Attitudes Toward ECG Interpretation (n = 100)

| **Statement** | **Disagree (1–2) n (%)** | **Neutral (3) n (%)** | **Agree (4–5) n (%)** |
| --- | --- | --- | --- |
| ECG interpretation is difficult | 8 (8.0) | 13 (13.0) | 79 (79.0) |
| ECG skills are not very important for ICU nurses | 89 (89.0) | 7 (7.0) | 4 (4.0) |
| Nurses should rely on physicians for ECG interpretation | 38 (38.0) | 35 (35.0) | 27 (27.0) |
| ECG knowledge helps timely arrhythmia diagnosis | 1 (1.0) | 3 (3.0) | 96 (96.0) |
| I have insufficient ECG knowledge | 22 (22.0) | 29 (29.0) | 49 (49.0) |
| ECG interpretation is not a nursing priority | 72 (72.0) | 17 (17.0) | 11 (11.0) |
| Monitoring vital signs is more important than ECG | 10 (10.0) | 19 (19.0) | 71 (71.0) |
| ECG interpretation is not a nurse’s responsibility | 77 (77.0) | 16 (16.0) | 7 (7.0) |
| Ongoing ECG education improves nurses’ skills | 2 (2.0) | 3 (3.0) | 95 (95.0) |
| I am not well trained to interpret ECGs during patient care | 20 (20.0) | 30 (30.0) | 50 (50.0) |

**Composite attitude score (10 items):** Median= 34.0, IQR= 4.75 (Cronbach’s α = 0.87).

Note. Responses were measured on a 5-point Likert scale (1 = Strongly Disagree, 5 = Strongly Agree). Categories were grouped into Disagree (1–2), Neutral (3), and Agree (4–5) for clarity.
