# Supplementary material for: Knowledge and Attitudes Towards ECG Interpretation Among Intensive Care Nurses: A Greek Cross‐Sectional Study
Source: Nurs Crit Care. 2026 Apr 25;31:e70505. doi: 10.1111/nicc.70505 (PMC13109745; doi:10.1111/nicc.70505)
Supplement: Supplementary file 2 — Table S2: Significant associations between ICU nurses' attitudes towards ECG interpretation and professional characteristics (n = 100). [file NICC-31-0-s003.docx]

# Supplementary Tables

Table S2. Significant Associations Between ICU Nurses’ Attitudes Toward ECG Interpretation and Professional Characteristics (n = 100)

| **Attitude Statement** | **Associated Factor** | **Test Statistic** | **p-value** | **Effect Size** |
| --- | --- | --- | --- | --- |
| “ECG interpretation is difficult” | ICU Experience | χ² = 18.826 | 0.020 | Cramer’s V = 0.29 |
| “ECG interpretation is difficult” | Frequency of ECG Evaluation | χ² = 14.831 | 0.022 | V = 0.27 |
| “I have insufficient ECG knowledge” | Unit Type | χ² = 13.241 | 0.010 | Phi = 0.26 |
| “I have insufficient ECG knowledge” | Years of Experience | χ² = 17.295 | 0.027 | V = 0.28 |
| “I have insufficient ECG knowledge” | ICU Experience | χ² = 18.254 | 0.019 | V = 0.29 |
| “ECG interpretation is not a nursing priority” | Unit Type | χ² = 13.475 | 0.009 | Phi = 0.27 |
| “ECG interpretation is not a nursing priority” | Frequency of ECG Evaluation | χ² = 23.591 | 0.000 | V = 0.35 |
| “Vital signs monitoring is more important than ECG” | Unit Type | χ² = 11.463 | 0.022 | Phi = 0.25 |
| “Vital signs monitoring is more important than ECG” | ICU Experience | χ² = 14.127 | 0.012 | V = 0.27 |
| “I am not well trained for ECG interpretation” | Unit Type | χ² = 9.256 | 0.009 | Phi = 0.23 |
| “I am not well trained for ECG interpretation” | Years of Experience | χ² = 15.818 | 0.045 | V = 0.24 |
| “I am not well trained for ECG interpretation” | ICU Experience | χ² = 20.085 | 0.010 | V = 0.28 |

**Composite attitude score (10 items):** Median= 34.0, IQR= 4.75 (Cronbach’s α = 0.87).

Note. Only statistically significant associations are reported (p < 0.05). Effect sizes are reported using Phi or Cramer’s V as appropriate. Non-significant results are omitted for clarity.
