# Supplementary material for: Knowledge and Attitudes Towards ECG Interpretation Among Intensive Care Nurses: A Greek Cross‐Sectional Study
Source: Nurs Crit Care. 2026 Apr 25;31:e70505. doi: 10.1111/nicc.70505 (PMC13109745; doi:10.1111/nicc.70505)
Supplement: Supplementary file 3 — Table S3: Summary of significant associations between ECG knowledge and attitudes and professional characteristics (n = 100). [file NICC-31-0-s001.docx]

# Supplementary Tables

Table S3. Summary of Significant Associations Between ECG Knowledge and Attitudes and Professional Characteristics (n = 100)

| **Outcome** | **Associated Factor** | **Direction of Association** | **Test Statistic** | **p-value** | **Effect Size** |
| --- | --- | --- | --- | --- | --- |
| **Knowledge Score** | ICU Type (Cardiac vs. General) | Higher knowledge in Cardiac ICU nurses | χ² = 9.952 | 0.002 | Phi = 0.32 |
|  | Prior ECG Training  (Yes vs. No) | Higher knowledge with prior training | χ² = 6.836 | 0.009 | V = 0.26 |
|  | Years of Experience  (>20 vs. ≤20) | Higher knowledge with >20 years clinical exp. | χ² = 11.240 | 0.024 | Phi = 0.27 |
| **Attitudes** | ICU Experience | Longer ICU exp. associated with higher difficulty perception & training needs | χ² = 18.826 / 20.085 | 0.020 / 0.010 | V = 0.28–0.29 |
|  | Years of Experience | More experienced nurses reported insufficient knowledge | χ² = 17.295 | 0.027 | V = 0.28 |
|  | Unit Type | Nurses in General ICUs more often rated ECG as “not a priority” | χ² = 13.475 | 0.009 | Phi = 0.27 |
|  | Frequency of ECG Evaluation | Frequent evaluators perceived ECG as more important | χ² = 23.591 | 0.000 | V = 0.35 |
|  | Vital Signs vs. ECG | General ICU nurses prioritized vital signs over ECG | χ² = 11.463 | 0.022 | Phi = 0.25 |

**Composite knowledge score (10 items):** Median= 6.0, IQR= 2.0, Cronbach’s α = 0.81.
**Composite attitude score (10 items):** Median= 34.0, IQR= 4.75, Cronbach’s α = 0.87.

Note. Only significant associations (p < 0.05) are presented. Effect sizes are reported as Phi or Cramer’s V. Full item-level results are available upon request.
